# Efficacy and safety of EBUS‐TBNA under conscious sedation with meperidine and midazolam

**DOI:** 10.1111/1759-7714.14286

**Published:** 2022-01-07

**Authors:** Roberto Piro, Eleonora Casalini, Matteo Fontana, Carla Galeone, Patrizia Ruggiero, Sofia Taddei, Giulia Ghidoni, Giulia Patricelli, Nicola Facciolongo

**Affiliations:** ^1^ Pulmonology Unit, Department of Medical Specialties Azienda Unità Sanitaria Locale ‐ IRCCS Tecnologie, Avanzate e Modelli Assistenziali in Oncologia di Reggio Emilia Reggio Emilia Italy; ^2^ Pulmonology Unit Azienda Sanitaria Locale Brindisi Brindisi Italy

**Keywords:** bronchoscopy, EBUS‐TBNA, meperidine, midazolam, sedation

## Abstract

**Background:**

According to the guidelines, endobronchial ultrasound guided transbronchial needle aspiration (EBUS‐TBNA) is the technique of choice for the diagnosis of mediastinal involvement in lung cancer; it is also useful for other mediastinal malignancies and benign pathology. Nevertheless, there is still discussion about whether to perform it under general anesthesia or under conscious sedation.

**Methods:**

We retrospectively analyzed the data of all patients who underwent EBUS‐TBNA under conscious sedation with up to 1 mg/kg of meperidine and up to 0.15 mg/kg of midazolam in the Interventional Pulmonology Unit of the Azienda USL‐IRCCS Santa Maria Nuova of Reggio Emilia during 2 consecutive years. Demographic data, indication for the procedure, duration, number of lymph node sampled, number of passes per station, diagnostic yield, drugs dosage, questionnaire score, and complications were collected.

**Results:**

A total of 302 patients underwent EBUS‐TBNA, and 68% of the patients were males and the mean age was 65 ± 13 years old. The average duration of procedures was 24.4 minutes and the mean dosage of drugs was 4.32 ± 1.52 mg for midazolam and 50.86 ± 13.71 mg for meperidine. The mean number of lymph nodes sampled per patient was 1.75 ± 0.82, and each patient received an average of 4.71 ± 1.78 passes. A total of 90.7% of patients completed the procedures, 85% had adequate samples, and 94.4% of patients declared with Likert's questionnaire that they strongly agree to repeat the test if necessary.

**Conclusion:**

EBUS‐TBNA performed under conscious sedation with meperidine and midazolam is feasible and well‐tolerated and has a similar diagnostic yield of that reported in literature.

## Graphical abstract

In this study involving 302 patients, EBUS‐TBNA performed under conscious sedation with meperidine and midazolam led to 85% exams with adequate samples, of whom 62% were diagnostic and 38% showed normal lymph node tissue. A total of 12% were inadequate and 3% of scheduled exams were not performed. Severe and persistent adverse events have not been observed. A total of 94.4% of patients declared that they strongly agree to repeat the exam if necessary.

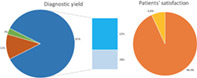

## INTRODUCTION

Endobronchial ultrasound guided transbronchial needle aspiration (EBUS‐TBNA) is an important tool for the diagnosis of mediastinal or hilar lymphadenopathy^1^, ^2^, ^3^ and lung cancer staging^4^, ^5^ because of its high yield, few complications, and low costs.^6^

Diagnostic precision of EBUS‐TBNA is compared to mediastinoscopy for staging of mediastinal lymph node.^7^, ^8^, ^9^ With linear EBUS, it is generally possible to sample the para‐tracheal (2 and 4), subcarinal (7), hilar and interlobar (10 and 11) lymph node stations. The limitation of EBUS in reaching certain sites (lymph node stations 8 and 9, as well as celiac axis, liver, and left adrenal gland) can be overcome by endoscopic ultrasound (EUS) or by endoscopic ultrasound‐bronchoscopy (EUS‐B), techniques in which trans‐esophageal samplings are performed respectively with eco‐gastroscope or with eco‐bronchoscope.^10^

Echo‐endoscopy allows to obtain tissue samples sufficient for cytohistological diagnosis, including immunohistochemical evaluation,^6^ and to avoid more invasive maneuvers (e.g., mediastinoscopy). Sedation protocols are very important in EBUS‐TBNA because they could modify diagnostic yield, duration of procedure, patient's satisfaction, and complications. First, studies on EBUS‐TBNA were performed under general anesthesia (GA) or deep sedation.^11^, ^12^ In this way, a high grade of depression of consciousness is achieved and it generally requires an anesthesiologist. Indeed, in some countries (e.g., in Italy) some drugs used in GA, such as propofol, cannot be administered by other physicians. Following studies showed that moderate sedation that can be managed by the interventional pulmonologist directly was an adequate approach^13^, ^14^ and EBUS‐TBNA performed with this approach have a similar diagnostic yield and rate of complications compared with those performed under GA.^15^ In this way, the pulmonologist plays a double role in both performing EBUS‐TBNA procedures and monitoring the sedation. Furthermore, there are two other issues that need consideration. First, it has been documented the cost‐reduction of EBUS‐TBNA performed under conscious sedation compared with GA^16^, ^17^; in the field of the pandemic we are currently living in, this is an important matter of concern.^18^ Second, the anesthesiologist's availability in contrast with the increasing number of EBUS‐TBNA procedures requested is critical in some hospitals; consequently, there is a strong need for pulmonologists to perform the procedures using strategies different from GA. Currently, the optimal sedation protocol for EBUS‐TBNA is still a matter of discussion.^17^, ^19^

The aim of this study was to assess efficacy and safety of EBUS‐TBNA under conscious sedation with meperidine and midazolam performed by pulmonologists, in terms of complete sampling of lymph nodes after consulting radiological imaging, diagnostic yield, and patient's satisfaction. The choice of these drugs is related with the current evidences: midazolam is the most used and recommended drug for conscious sedation during flexible bronchoscopy,^20^, ^21^ whereas meperidine, although discussed,^22^ has been widely investigated and used both in bronchoscopic procedures^23^ and in digestive endoscopic procedures^24^, ^25^ with good results.

## MATERIALS AND METHODS

### Ethics statement

This study was approved by the local institutional review board. The protocol was performed according to Good Clinical Practice (International Council for Harmonisation of Technical Requirements for Pharmaceuticals for Human Use ‐ ICH ‐ Harmonized Tripartite Guidelines for Good Clinical Practice 1996; Directive 91/507. EEC, The Rules Governing Medical Products in the European Community) and Italian laws.

### Study population and procedure

This retrospective study analyzed data of all patients that underwent EBUS‐TBNA and referring to the Interventional Pulmonology Unit of Azienda USL di Reggio Emilia/IRCCS in Reggio Emilia, Italy, for 2 consecutive years. Data were analyzed in 2020. Patients enrolled in this study were older than 18 years, were able to sign informed consent, and had a positive chest computed tomography (CT) or positron emission tomography (PET). They underwent procedures for diagnosis and/or staging of mediastinal or hilar lymphadenopathies or paratracheal or peribronchial masses (positive PET or short axis ≥1 cm). In this work, we considered only patients who underwent procedures with selective lymph nodes sampling (e.g., 1‐3 lymph nodes). Exclusion criteria included pregnancy, low platelet count, tachycardia, chronic renal failure, history of sedation drugs allergy, and alcoholism.

Before every procedure, the pulmonologist identified the sampling scheme for each lymph node to perform EBUS‐TBNA. Local anesthesia was performed before the administration of sedative agents to minimize patient's discomfort with 300 mg of lidocaine 2%, dispensed first with a manual nebulizer over the vocal cords and second with a 10 mL syringe educating the patient to gargle before swallowing the local anesthetic. During bronchoscopy, vital signs (pulse oximetry, respiratory rate, and blood pressure), continuous electrocardiogram, and chest excursions were monitored according to British Thoracic Society guidelines.^20^

We performed procedures under conscious sedation with intravenous meperidine and midazolam: initial induction dose of meperidine ranging 15–50 mg (intake to bodyweight) with a slow intravenous bolus, following 5–10 mg bolus with a limit of 25–30 mg at levels up to 1 mg/kg; initial induction dose of midazolam ranging 1–2 mg with a slow intravenous bolus, according to effects (slowdown of speech, relaxation of facial muscles) following 1–1.5 mg bolus administered to obtain appropriate sedation, at levels up to 0.15 mg/kg.

EBUS‐TBNA was performed orally with bronchoscope (BF‐UC180OF Olympus) using a dedicated 22‐gauge needle. After ultrasound examination, transbronchial punctures were performed with at least three needle passes for each lymph node according to the sample scheme identified before each procedure. The collected material was smeared on glass slides and fixed in formalin for cytological analysis. Rapid on‐site evaluation (ROSE) was not performed. Approximately 1 to 2 hours after procedures, Likert's questionnaire was administered with the question “would you agree to repeat the procedure if it was necessary?” to value patient's satisfaction about the procedure. The subject could answer with five statements: strongly disagree, disagree, uncertain, agree, and strongly agree. After this time of observation, patients were discharged.

### Data and statistical analysis

For each patient we collected demographic data (age, gender, and weight), indication for EBUS‐TBNA, procedure duration, number of lymph node sampled per patient, number of passes per station, diagnostic yield, dose of drugs used for sedation, questionnaire score, and complications (cough, tachyarrhythmia, hypotension, hypertension, hemorrhage, desaturation, difficult to sedate, and laryngospasm).

Results were divided in quantitative and qualitative data; the first were indicated as mean and standard deviation (SD), whereas the second were indicated as number and percentage.

## RESULTS

Overall, 302 patients underwent EBUS‐TBNA at the Interventional Pulmonology Unit of Azienda USL di Reggio Emilia‐IRCCS and were enrolled in the study. Among these, 204 (68%) were male and mean age was 65 ± 13 years old (Table 1). Main indications for EBUS‐TBNA were pulmonary or pleural diseases and multiple lymphadenopathies detected with radiological investigations (172 patients, 57%), isolated mediastinal lymphadenopathy (110 patients, 36%), and peribronchial masses (20 patients, 12%) (Table 1).

**TABLE 1:** Baseline patient characteristics and indications for EBUS‐TBNA

Patient characteristics	
No. of patients	302
Age, mean ± SD, y	65 ± 13
Male, n (%)	204 (68)
Weight kg, mean ± SD	71.3 ± 13.6
Indications for EBUS‐TBNA	
Pleural or pulmonary diseases and lymphadenopaties, n (%)	172 (57)
Isolated mediastinal lymphadenopaty, n (%)	110 (36)
Peribronchial mass (T), n (%)	20 (12)

Abbreviation: T, tumor.

The average duration of procedures was 24.4 minutes. The mean dosage of drugs was 4.32 mg (SD 1.52 mg) for midazolam and 50.86 mg (SD 13.71 mg) for meperidine. Overall, 502 lesions/lymph node stations were sampled; subcarinal station (no. 7: 166 samples, 33%) and right lower paratracheal station (no. 4R: 139 samples, 27%) were the most frequent stations investigated. The mean number of lymph nodes sampled per patient was 1.75 (SD = 0.82) and each patient received at average 4.71 passes (SD = 1.78) (Table 2).

**TABLE 2:** EBUS‐TBNA procedure features

EBUS‐TBNA procedure	
Procedure duration, min mean ± SD	24.36 ± 6.23
Sedation	
Meperidine, mg mean ± SD	50.86 ± 13.71
Midazolam, mg mean ± SD	4.32 ± 1.52
Lymph nodes stations sampled	
Total lymph nodes, n	502
1, n (%)	1 (0.4)
2R, n (%)	13 (3)
3, n (%)	2 (0,6)
4R, n (%)	139 (27)
4L, n (%)	49 (10)
7, n (%)	166 (33)
10R, n (%)	9 (2)
10L, n (%)	7 (1)
11R, n (%)	65 (13)
11L, n (%)	26 (5)
12 R, n (%)	6 (1)
T, n (%)	18 (4)
No. of lymph nodes sampled per patient, n mean ± SD	1.75 ± 0.82
No. of passes per patient n mean ± SD	4.71 ± 1.78

Abbreviation: T, tumor.

Among the group of 302 patients, 274 (90.7%) completed the procedures, whereas for 27 patients, EBUS‐TBNA was stopped prematurely for complications (described in Table 3; cough, tachyarrhythmia, hypotension, hypertension, hemorrhage, desaturation, difficult to sedate, and laryngospasm). In particular, two patients experienced severe oxyhemoglobin desaturation (desaturation of ≥10 points from basal), and five patients presented tachyarrhythmia with rapid and spontaneous resolution. The procedure was not performed at all in only one patiet because of a complication (marked hypertension occurred during the ultrasound examination).

**TABLE 3:** Complications

Complications	
Adverse events total, n	28
Tachyarrhythmia, n (%)	5 (2)
Hypotension	1 (0)
Hypertension, n (%)	3 (1)
Severe peripheral SpO_2_ desaturation, n (%)	2 (1)
Difficult to sedate, n (%)	6 (2)
Cough, n (%)	7 (2)
Hemorrhage, n (%)	1 (0)
Laryngospasm, n (%)	2 (1)
Sick, n (%)	1 (0)

Procedures were repeated with general anesthesia in only seven patients (2%).

**FIGURE 1:**
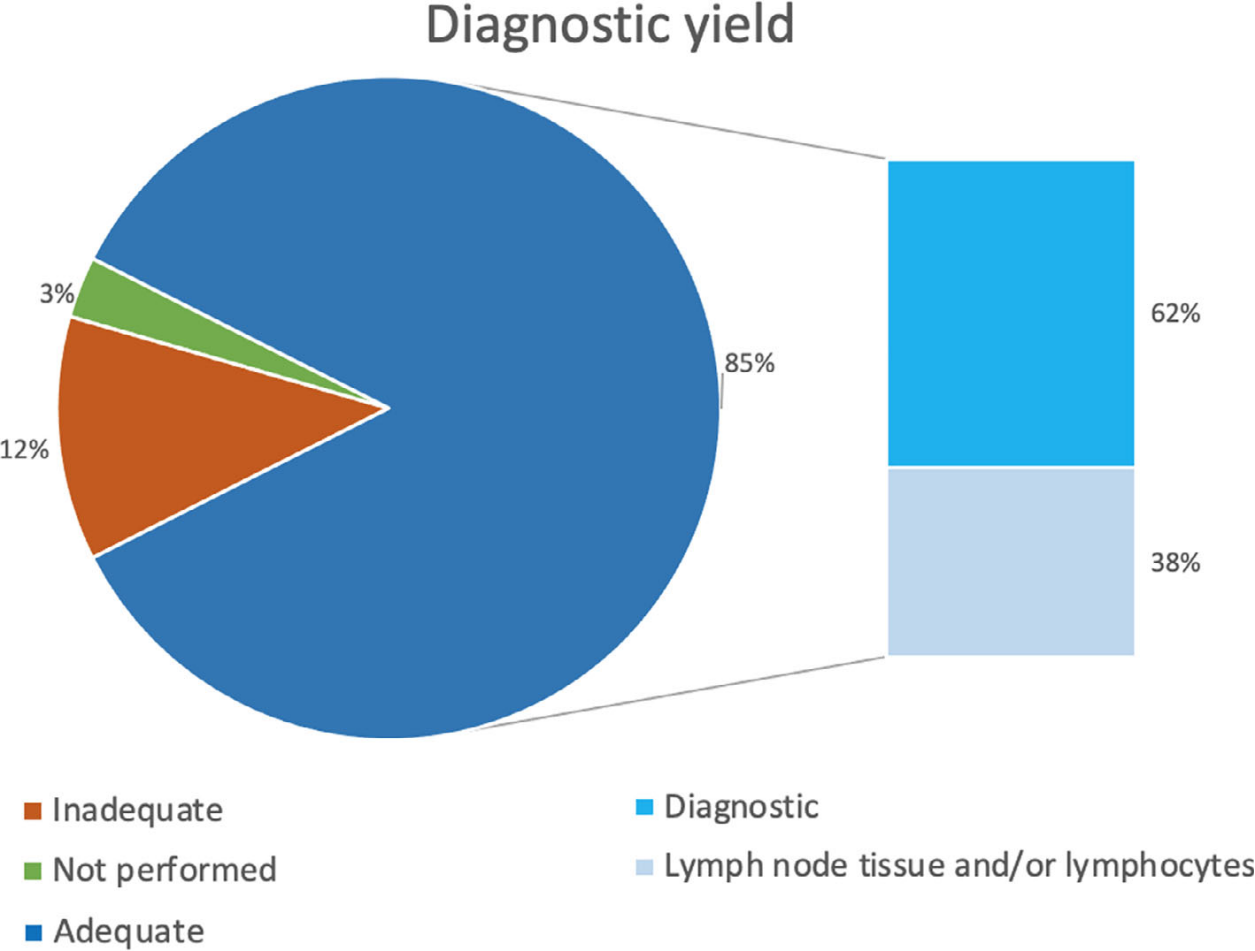
EBUS‐TBNA diagnostic yield. A total of 12% of the procedures showed inadequate sampling, 3% were not performed, and 85% showed adequate sampling, which 62% were diagnostic for cancer and 38% were negative for tumor cells, but containing lymph node tissue or lymphocytes

Among 302 procedures included in this study, 85% had adequate samples (among this, 62% was diagnostic, whereas 38% contained normal lymph node tissue and/or lymphocytes), 12% had inadequate samples and 3% were not performed because the expected lymph nodes were not detectable on ultrasound. (Figure 1). A total of 128 patients (42.3%) were diagnosed with lung cancer, 31 patients (10%) with granulomatous diseases, and one patient (0.3%) with tuberculosis. On the other hand, in 133 patients (44.4%) samplings resulted negative for malignancy or were consistent with reactive lymph nodes (Table 4).

**TABLE 4:** EBUS‐TBNA diagnosis (in 3% patients [9], EBUS‐TBNA was not performed)

Final diagnosis	
Lung cancer, n (%)	128 (42.3)
Granulomatous disease, n (%)	31 (10)
Tuberculosis, n (%)	1 (0.3)
Reactive lymph node/negative for malignancy, n (%)	133 (44.4)

At the end of the procedures, as assessed by Likert's questionnaire, 285 patients (94.4%) declared that they strongly agree to repeat the test if necessary, whereas only two patients strongly disagree with it.

## DISCUSSION

Assuming that EBUS‐TBNA can be performed under either general anesthesia or conscious sedation,^26^ recent studies were focused on the last option,^14^, ^19^, ^27^, ^28^ to improve the work flow of endoscopic procedures, to perform it without the presence of an anesthesiologist and to reduce costs.

To date, only one randomized study^14^ compared the two methods of sedation and it showed no difference between both sedations in terms of diagnostic yield, major complications, and patient satisfaction.

Other studies demonstrated that the choice of sedation method did not impact on EBUS‐TBNA outcomes^15^; in addition to this, Boujaoude et al.^17^ showed that moderate sedation is related to a minor impact on costs for the health system.

According to available data, we managed an observational retrospective study collecting data about patients that underwent EBUS‐TBNA in conscious sedation, referring to our Interventional Pulmonology Unit.

Meperidine was chosen, compared with other opioids, because of the plasmatic half‐life of the drug, greater than the one of fentanyl or alfentanyl, which could result in a more stable level of sedation during the procedure without the need of adding other drugs, as in our experience.^29^ Furthermore, another secondary reason was the expertise of our group with this pharmacological agent, which has been used for several years without the observation of severe adverse events, as showed in this study. The good safety profile of meperidine compared with fentanyl is also well‐documented in other studies on gastrointestinal endoscopy comparing these two agents.^25^, ^30^

The planned sample scheme was completed in 90.7% of procedures; nevertheless, in 97% of the total procedures at least a sample was obtained, whereas in 85% cases samples were adequate. This result is more significant than other reports in different studies,^31^ supporting the efficacy of conscious sedation in terms of accomplishment of the scheduled procedure and diagnostic yield. In 62% of adequate procedures, we obtained specific diagnosis, in line with other studies.^32^, ^33^ We currently do not have many available data analyzing the impact of different sedation methods on EBUS‐TBNA diagnostic yield, even if this procedure is a deeply studied subject.^14^, ^15^, ^34^

Previous studies showed that the sedation method applied did not influence the satisfaction of patients that underwent procedures.^13^, ^14^ In the Steinfort's study, 98% of patients achieved high satisfaction in conscious sedation with midazolam and fentanyl and the possible addition of propofol.^33^ According to them, the data we collected about satisfaction in our study were significant: 94.4% patients answered that they would repeat the procedure if necessary.

Results about complications during procedures in our cohort showed only 0.7% of patients experiencing severe oxyhemoglobin desaturation and 1.7% of patients presenting tachyarrhythmia, with spontaneous resolution. The most frequent complication was cough, observed in 2.3% of patients. Only 7 (2.3%) patients had to repeat the procedure with an anesthesiologist, who added propofol at the sedation protocol. These results appear to be similar compared with procedures performed with GA, where only major adverse events were measured.^35^, ^36^

Our study has some limitations. First, the retrospective nature of the study makes the results difficult to be generalizable. Second, this is a monocentric study, and for this reason, the expertise of a single interventional pulmonology team in the use of a specific sedative agent had a role, whereas the use of the same drug should be assessed also in other groups of specialists. Third, the lack of a control group does not allow drawing conclusions on the potential advantages of the sedation strategy with midazolam and meperidine over other strategies with different drugs. Fourth, in this work, we focused on only patients undergoing selective lymph nodes sampling (e.g., from 1‐3 lymph nodes sampled), whereas data on systematic EBUS‐TBNA staging, that is a longer and potentially more difficult procedure to perform under conscious sedation, are lacking in the present study. On the other hand, there are also strengths about this work, in particular regarding the number of the study population (302 patients) and the outcomes measured (diagnostic yield, safety, and patient satisfaction).

## CONCLUSION

This retrospective study supports the efficacy, tolerability, and safety of conscious sedation with midazolam and meperidine in EBUS‐TBNA. Conscious sedation is a very important tool for interventional pulmonologists because it obtains satisfying outcomes with lower resources by combining sedative and amnesic properties of benzodiazepines with anticough and analgesic properties of opioids.

As this study included patients who underwent selective lymph node sampling, further studies are needed to compare the use of conscious sedation and general anesthesia in EBUS‐TBNA systematic staging of the mediastinum.

## DISCLOSURES

The authors declare no conflicts of interest.
